# Correlation between dielectric property by dielectrophoretic levitation and growth activity of cells exposed to electric field

**DOI:** 10.1007/s00449-012-0849-3

**Published:** 2012-11-20

**Authors:** Masaru Hakoda, Yusuke Hirota

**Affiliations:** Department of Chemical and Environmental Engineering, Gunma University, 1-5-1 Tenjin Cho, Kiryu, Gunma 376-8515 Japan

**Keywords:** Dielectrophoresis, AC electric field, DC electric field, Analytical activity

## Abstract

The purpose of this study is to develop a system analyzing cell activity by the dielectrophoresis method. Our previous studies revealed a correlation between the growth activity and dielectric property (Re[*K(ω)*]) of mouse hybridoma 3-2H3 cells using dielectrophoretic levitation. Furthermore, it was clarified that the differentiation activity of many stem cells could be evaluated by the Re[*K(ω)*] without differentiation induction. In this paper, 3-2H3 cells exposed to an alternating current (AC) electric field or a direct current (DC) electric field were cultivated, and the influence of damage by the electric field on the growth activity of the cells was examined. To evaluate the activity of the cells by measuring the Re[*K(ω)*], the correlation between the growth activity and the Re[*K(ω)*] of the cells exposed to the electric field was examined. The relations between the cell viability, growth activity, and Re[*K(ω)*] in the cells exposed to the AC electric field were obtained. The growth activity of the cells exposed to the AC electric field could be evaluated by the Re[*K(ω)*]. Furthermore, it was found that the adverse effects of the electric field on the cell viability and the growth activity were smaller in the AC electric field than the DC electric field.

## Introduction

Dielectrophoresis (DEP) is the motion of cells caused by polarization effects in a nonuniform electric field. The cells move to the lower electric field or the higher electric field side due to the relationship between the permittivity of the cells and the permittivity of the medium. In studies using DEP, separation and manipulation of cells etc. have been mainly performed, for example, the separation of viable and nonviable cells from their mixture [1–10], separation of specific cells from a cell suspension in which several species of cells were mixed [11, 12], and cell manipulation using the difference in electrode geometry [13]. Moreover, it was reported that the cells’ exposure to electric fields has no detrimental effect on viability, cell growth, and metabolism [14, 15]. Thus, DEP is a very effective method for the separation and manipulation of cells.

Although viable and nonviable cells have been separated and types of cells have been separated using dielectrophoresis in several studies, there is little research on the analysis of the activity of a single cell. Regarding the analysis method of the cell activity, measurement of the cell growth rate and the cell sorter is mainly used. However, those analytical methods either take several days or the reagent for analysis, and the analyzer is very expensive. Moreover, the cells used for the analysis cannot be used for cultivation or for another experiment.

The advantage of DEP levitation is the low cost of the measurement apparatus and the immediacy of the analysis results. In studies using DEP levitation, it has been reported that the dielectric property differs by changing the pH and the conductivity of the medium at the protoplast of plant cells [16, 17], and the correlation between the growth activity and the dielectric property in mouse hybridoma 3-2H3 cells was obtained [18]. Moreover, in our previous studies [19, 20], we discussed the possibility of measuring the differentiation activity for rat mesenchymal stem cells (RMSC), human mesenchymal stem cells (HMSC), and human adipose tissue-derived stem cells (ASC) by DEP levitation. Consequently, it was found that the differentiation activity of those stem cells could be evaluated by DEP levitation.

In the present study, an animal cell exposed to an alternating current (AC) electric field or a direct current (DC) electric field was cultivated, and the influence of the electric field on the growth activity of the cell was examined. Furthermore, the system analyzing cell activity by measuring the dielectric property was examined by verifying the correlation between the dielectric property and the growth activity of the cells exposed to the electric field.

## Theory

In a nonuniform AC electric field, a cell moves to a high electric field or a lower electric field by the relationship between the permittivity of the cell and the permittivity of the medium. The cell moves toward the higher electric field when the permittivity of the cell is higher than that of the medium (positive DEP). Conversely, the cell moves toward the low electric field when the permittivity of the cell is lower than that of the medium (negative DEP). The DEP force is given by the following equation:

1 $$ F_{DEP} = 2\pi r^{3} \varepsilon_{m} \text{Re} \left[ {K\left( \omega \right)} \right]\nabla E^{2} $$

where *r* is the cell radius, *ε*_*m*_ is the permittivity of the medium, and *E* is the effective value of electric field intensity. The dielectric property (Re[*K(ω)*]) indicates the real part of the Clausius–Mossotti function and is given by the following equation:

2 $$ K\left( \omega \right) = \frac{{\varepsilon_{c}^{ * } - \varepsilon_{m}^{ * } }}{{\varepsilon_{c}^{ * } + 2\varepsilon_{m}^{ * } }} $$

where $\varepsilon_{c}^{ * }$ and $\varepsilon_{m}^{ * }$ are complex permittivity of the cell and the medium, respectively:

3 $$ \varepsilon_{c}^{ * } = \varepsilon_{c} - j\frac{{\sigma_{c} }}{\omega };\;\;\varepsilon_{m}^{ * } = \varepsilon_{m} - j\frac{{\sigma_{m} }}{\omega } $$

where $\varepsilon_{c}^{{}}$ and $\sigma_{c}^{{}}$ are the permittivity and conductivity of the cell, respectively and $\varepsilon_{m}^{{}}$ and $\sigma_{m}^{{}}$ are the permittivity and conductivity of the medium, respectively.

At the DEP levitation, the cell is stably levitated by balancing the downward directed gravitational force and the upward directed force produced by buoyance and positive DEP. In this case, the equation is given by the following:

4 $$ \frac{4}{3}r^{3} \pi \rho_{c} g = \frac{4}{3}r^{3} \pi \rho_{m} g + 2\pi r^{3} \varepsilon_{m} \text{Re} \left[ {K\left( \omega \right)} \right]\nabla E^{2} $$

where *ρ*_*c*_ is mass density of the cell and *ρ*_*m*_ is density of the medium. Equation (4) simplifies to

5 $$ \text{Re} \left[ {K\left( \omega \right)} \right] = \frac{2}{3} \cdot \frac{{g\left( {\rho_{c} - \rho_{m} } \right)}}{{\varepsilon_{m} \nabla E^{2} }} .$$

By the cell being stably levitated, Eq. (5) is independent on the cell size. Thus, Re[*K(ω)*] is defined as the dielectric property of the cell, and is examined as index of the cell activity evaluation.

**Fig. 1:**
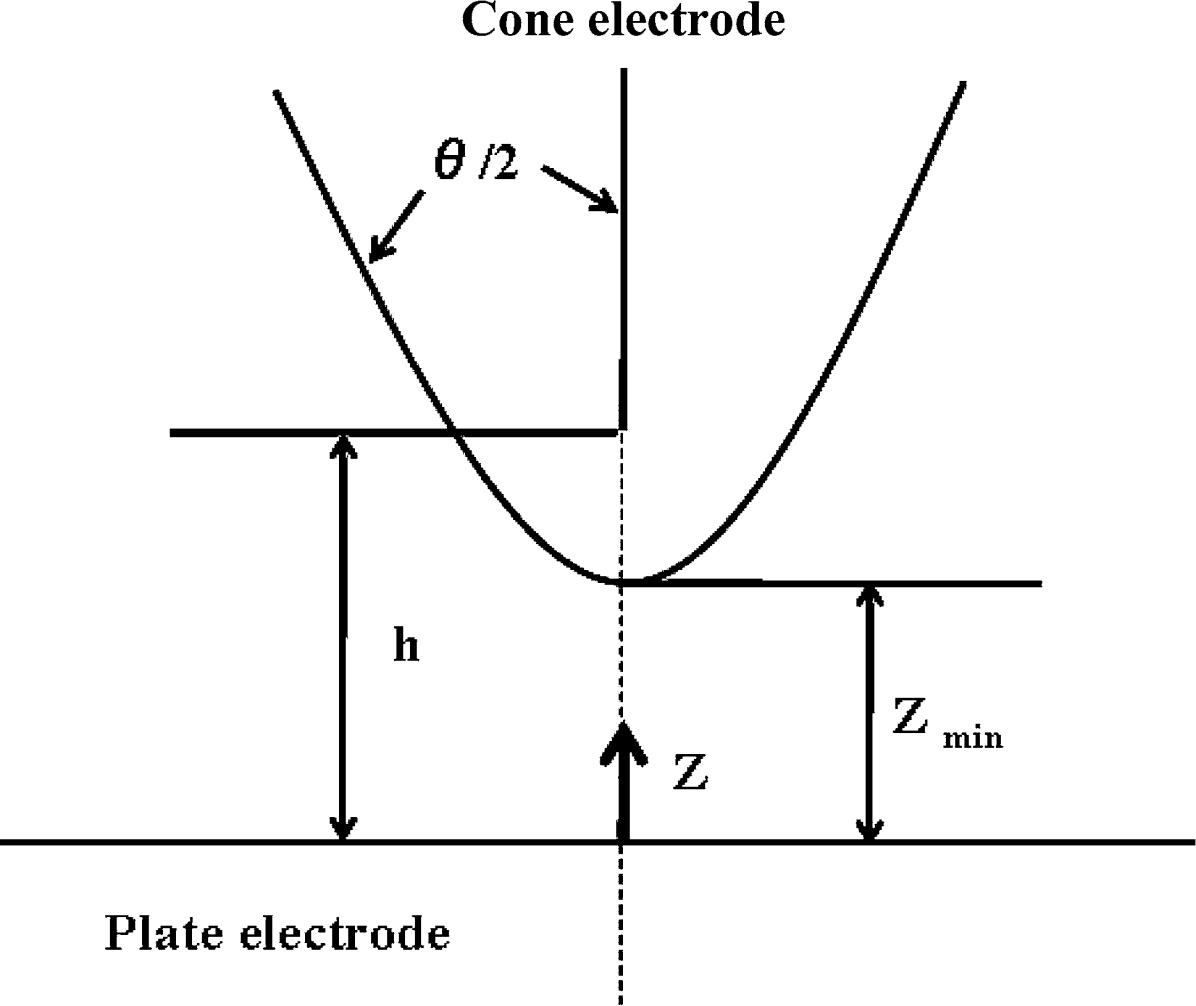
Schematic diagram of cone-plate type electrode

$\nabla$ *E* ^*2*^ in the static position *z* of the cell from the plate electrode shown in Fig. 1 is calculated from Eqs. (6), (7).

6 $$ \nabla E^{2} \left( z \right) = \frac{{16V_{0}^{2} h^{2} z}}{{\left( {h^{2} - z^{2} } \right)^{3} \left\{ {\ln \left( {\frac{{h + z_{\hbox{Min} } }}{{h - z_{\hbox{Min} } }}} \right)} \right\}^{2} }} $$

7 $$ h = \frac{{z_{min} } }{{\cos \left( {\frac{\theta }{2}} \right)}} $$

Where *V*_*0*_ is the applied voltage and *z*_*min*_ is distance of the electrode spacing, *θ* is the asymptotic cone angle [21].

## Materials and methods

### Cell cultivation

In this experiment, mouse hybridoma 3-2H3 cells (RCB0867, Riken Cell Bank, Japan) were used as the sample cell. The growth medium for the cells was DMEM (D6429 Sigma) supplemented with 10 % FBS, 100 units/mL penicillin G potassium (Banyu Pharmaceutical Co., Ltd.) and 10 mg/L streptomycin (Meiji Seika Co., Ltd.) were also added. The cells were cultivated in an incubator (BNA-111, ESPEC Co.) at 5 % CO_2_ and 310 K.

### Sample preparation

The cells were centrifuged at 277 K at 1,500 rpm for 3 min. The cell pellets were washed and resuspended twice in an isotonic solution consisting of 8.5 % (w/v) sucrose plus 0.3 % (w/v) dextrose buffer. The cell concentration was measured using a hemocytometer (Improved Neubauer, Minato Medical Co., Ltd.) with a trypan blue stain of 0.4 % (15250, GIBCO). The nonviable cell is dyed by the trypan blue, and the viable cell is not dyed. The viability is defined by following equation:

8 $$ {\text{Viability}} = \frac{{X_{V} }}{{X_{V} + X_{NV} }} $$

where *X*_*V*_ and *X*_*NV*_ are the cell concentration of the viable cell and the nonviable cell, respectively.

### Electric field loading device

**Fig. 2:**
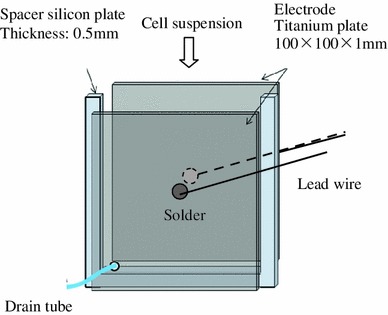
Schematic diagram of electric field loading device

An electric field loading device is shown in Fig. 2. A parallel plate electrode device was used to expose the sample cells to a uniform electric field. The device is made of pair titanium plate electrodes (100 × 100 × 1.0 mm) that sandwiched the silicon sheet with a thickness of 0.5 mm as a spacer. The shape and size of electric field loading device were necessary to get the high electric field and a lot of cells for the cultivation. The cell suspension was supplied in the device, and the electric field was applied. In the experiment that the cells were exposed to the electric fields, the electric field was applied for 10 min. The cell suspension in the device was circulated through a pipette to control the sedimentation of the cells. The device was immersed in an ice water tank to suppress the temperature rise. The medium temperature used was 278 K to prevent the deactivation of the cells.

### DEP levitation device

**Fig. 3:**
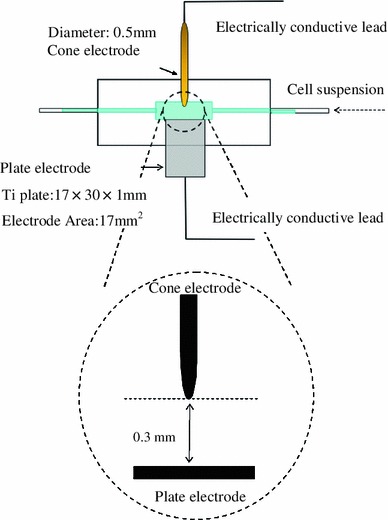
Schematic diagram of the experimental device of DEP leviation

A DEP levitation device for obtaining the Re[*K*(*ω*)] is shown in Fig. 3. The device is made of pair glass plates that sandwich the acrylic-resin plate with a thickness of 1 mm as a spacer. A cone electrode is made of stainless steel with a diameter of 500 μm and the angle at the tip is *θ* = 28°. A plate electrode is made of 1 mm thick titanium. The distance between the cone electrode and the plate electrode is 300 μm (*z*_*min*_) and *h* = 325 μm.

### Measurement of DEP levitations

**Fig. 4:**
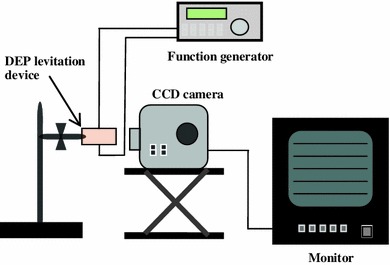
Schematic diagram of the experimental apparatus of DEP levitation

A schematic diagram of the experimental apparatus of the DEP levitation is shown in Fig. 4. The measuring method of the balancing position of a single cell is as follows [18].

- The cell suspension is filled in the DEP levitation device.
- The high voltage of 10 Vpp is applied to move a cell to the top of the corn electrode.
- The voltage is removed, and the cell is separated from the corn electrode by the gravitational force.
- A suitable voltage of 6.6 Vpp is applied, and the cell stays at the balancing position.

The cell suspension was injected into the DEP levitation device with a syringe. The movement of the cell on applying the AC voltage was observed by a CCD camera system (CCD MICROSCOPE Inf-500, Moritex Co., Japan and TRINITRON SONY Co., Japan). Watching the monitor, the cell was stably levitated by applying AC voltage with a function generator (33250A, Agilent Technologies Co., Ltd., USA). The distance from the plate electrode to the static position of the cell at that time was measured. The Re[*K*(*ω*)] was calculated by Eq. (5). In addition, the sedimentation rate of each cell was measured, and the mass density of the cell was calculated using the Stokes sedimentation rate equation. All experiments were carried out in a temperature controlled room at 298 ± 1 K.

### Measurement procedure of viability, growth activity and Re[*K*(*ω*)] changing by time progress

**Fig. 5:**
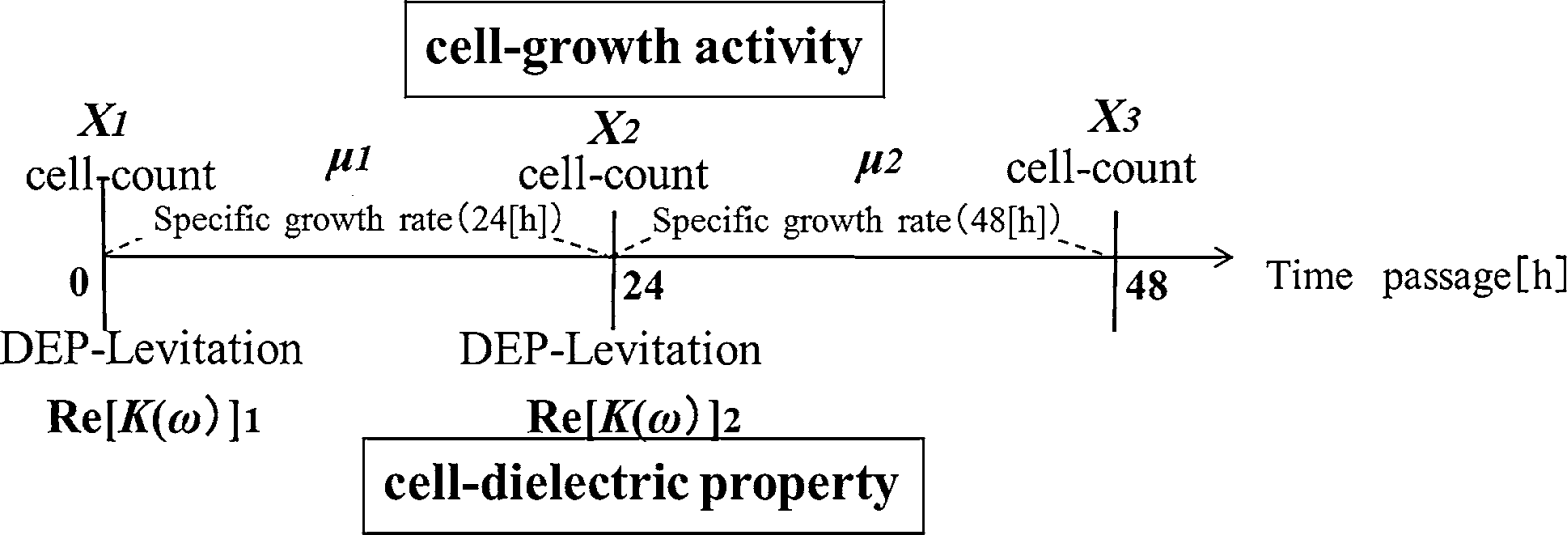
Flow chart of the experiment procedure

After cells were exposed to the electric field, the relationship between the viability, the cell activity and the Re[*K*(*ω*)] were investigated. The cell concentration and the Re[*K*(*ω*)] were measured with time course as follows. The flow chart of the experiment procedure was shown in Fig. 5.

1. The cells were exposed to the electric field and the concentrations of viable cells and nonviable cells were measured. The Re[*K(ω)*] of the cells exposed to the electric field was measured by the DEP levitation method. Afterwards, the cells exposed to the electric field were cultivated.
2. The concentration of cultivated cells up to 24 h was measured, and the Re[*K(ω)*] of the cells was measured at the same time. The specific growth rate was assumed to be the cell growth activity when its cultivation begun.
3. The concentration of cultivated cells up to 48 h was measured. The relation between the cell growth activity and the dielectric property Re[*K(ω)*] was examined.

The specific growth rate *μ* is defined by the following equation:

9 $$ \mu = \frac{{\ln \left( {{{X_{N} } \mathord{\left/ {\vphantom {{X_{N} } {X_{0} }}} \right. \kern-0pt} {X_{0} }}} \right)}}{N} $$

where *X*_0_ and *X*_*N*_ are the initial cell concentration and the cell concentration *N*th days, respectively.

## Results and discussion

### Effect of electric field on cell viability

**Fig. 6:**
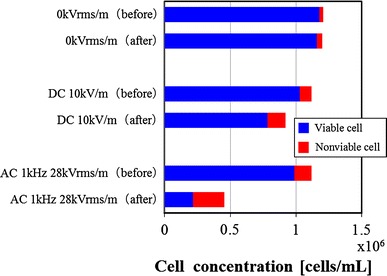
Effect of AC electric field stress or DC electric field stress on total cell concentration of 3-2H3 cells. (DC voltage: 10 kV/m, AC voltage: 1 kHz, 21 kVrms/m)

The changes in the cell concentration before and after the electric field stress load experiment to the 3-2H3 cell were examined. Examples of the change in the concentration of the viable and nonviable cell before and after the electric field stress load are shown in Fig. 6. The cell concentration before and after the stress load experiment under the condition of no electric field (0 kVrms/m) were a constant value. In the DC electric field (10 kV/m), the cell concentration decreased slightly, and the nonviable cells increased.

**Fig. 7:**
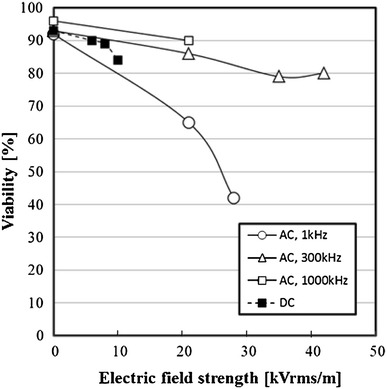
Effect of electric field strength on viability of 3-2H3 cells

In the AC electric field (1 kHz, 21 kVrms/m), the nonviable cells increased. The influence of electric field strength on the viability of the cells that received the electric field stress was examined. The influence of the electric field strength on the viability of the cells when the AC electric field of 1, 300 or 1,000 kHz, or the DC electric field was applied is shown in Fig. 7. In all experimental conditions, the viability decreased by increasing the electric field strength. Furthermore, the decreasing tendency of the viability of cells was dependent on the frequency of the AC electric field. At 1 kHz, which is the low frequency, the viability decreased dramatically by increasing the electric field strength. However, the viability was decreased slightly by increasing the electric field strength at 300 and 1,000 kHz. The viability of the DC electric field 10 kV/m was higher than that of 1 kHz in the AC electric field.

### Effect of cultivation on cell viability

To evaluate the growth activity of the cells exposed to the electric field stress, the cells were cultivated for 24 and 48 h.

The cell cultivation was performed in the following procedures.

- The cell suspension of the 3-2H3 is put in the electric field loading device, and the electric field stress is applied to the suspension for 10 min in the ice water bath.
- The suspension is moved to another vessel from the electric field loading device and cultivated in an incubator at 5 % CO2 and 310 K for cultivating the cells for 24 or 48 h without applying the electric field stress.
- After the cultivation, the trypan blue is put into the suspension and it is applied to a hemocytometer to examine the concentrations of viable cells and nonviable cells.

**Fig. 8:**
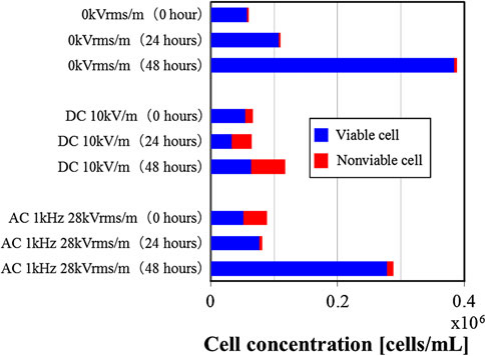
Effect of AC electric field stress or DC electric field stress on cells concentration after cultivated

**Fig. 9:**
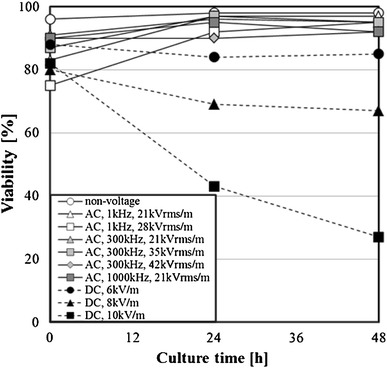
Effect of electric field strength on viability of 3-2H3 cells at culture time

Examples of the change in the cell concentration at the cultivation time are shown in Fig. 8. When the cells exposed to the DC electric field (10 kV/m) were cultivated for 48 h, the nonviable cells increased and the viable cells did not increase. On the other hand, the cells exposed to the AC electric field (1 kHz, 21 kVrms/m) grew well in the cultivation process, and the viable cells increased. Next, the cells exposed to the electric field were cultivated, and the relation between the viability and the cultivation time was examined. The experimental results are shown in Fig. 9. For the cells that were exposed to the AC electric field and cultivated, the viability was 90 % or more. On the other hand, the viability of the cells that were exposed to the DC electric field and cultivated decreased, as the cultivation time passed. It was revealed that damage to the cells exposed to the DC electric field increased with the progress of cultivation time. With the influence of the AC electric field on the cells, the viable cell concentration decreased and the nonviable cell concentration increased temporarily (Fig. 6). However, it was found that the adverse effect on the cells exposed to the AC electric field by the cultivation is almost lost. For the DC electric field, the viable cells hardly grew even if the cultivation operation was conducted, and the nonviable cell concentration increased. Therefore, it was verified that the use of the AC electric field was a more effective method than the DC electric field.

### Effect of electric field on cell growth activity

**Fig. 10:**
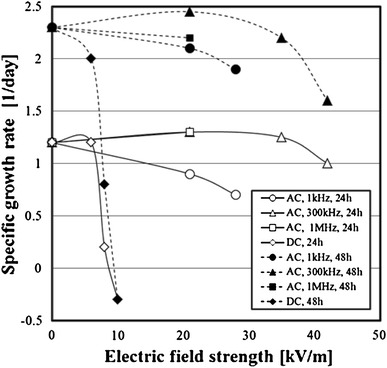
Effect of electric field on growth activity of 3-2H3 cells

Effects of the AC electric field or the DC electric field on the specific growth rate of the cells were examined. The specific growth rates after 24 and 48 h of the cells exposed to the electric field are shown in Fig. 10. Both specific growth rates after 24 and 48 h in cultivation of the cells exposed to the AC electric field decreased slightly compared with the cells not exposed to the electric field. At the frequency of 1 kHz, the specific growth rate decreased slightly by increasing the electric field strength. On the other hand, both the specific growth rates of the cells exposed to the DC electric field decreased dramatically after 24 and 48 h in cultivation. In addition, the specific growth rate was a negative value in the DC electric field 10 kV/m. In other words, the cells received the serious damage at the DC electric field 10 kV/m, and the cells died.

### Effect of electric field on dielectric property

**Fig. 11:**
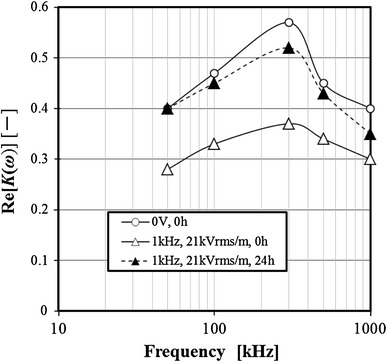
Effect of electric field strength on frequency property of Re[*K*(*ω*)]

**Fig. 12:**
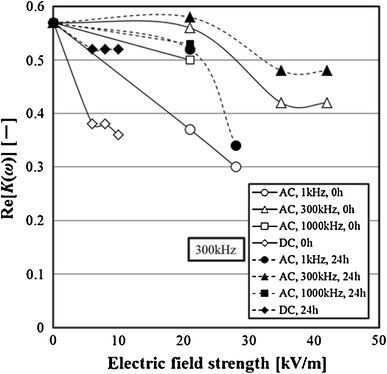
Effect of electric field on Re[*K(ω)*] of 3-2H3 cells

The influence of the electric field on the Re[*K(ω)*] frequency response of 3-2H3 cells was examined. Examples of the results are shown in Fig. 11. The value of Re [*K (ω)*] is an average value of the samples from 10 to 20 of the viable cells. All results of the frequency response of Re[*K(ω)*] showed a maximum value at the frequency of 300 kHz. Furthermore, although the Re[*K(ω)*] of the normal cells was large, the Re[*K(ω)*] of the cells immediately after exposure to the AC electric field was small. However, the Re[*K(ω)*] of the cells exposed to the electric fields were restored to the Re[*K(ω)*] of the normal cells by being cultured. Next, each influence of the AC electric field or the DC electric field on Re[*K(ω)*] of the cells was examined. The experimental results are shown in Fig. 12. From the results of Fig. 10, the Re[*K(ω)*] indicated the maximum value at 300 kHz. Therefore, the Re[*K(ω)*] at the frequency of 300 kHz was used and examined. In both the AC electric field and the DC electric field, the Re[*K(ω)*] of the exposed cells to the electric field decreased by increasing the electric field strength. In each experiment, the effect of the electric field strength on the Re[*K(ω)*] was similar to the effect of the electric field strength on the specific growth rate.

Moreover, in the both cases of AC electric field and DC electric field, the Re[*K(ω)*] of the cultivated cells was bigger than that of the cells exposed to the electric field.

### Relation between viability and dielectric property

**Fig. 13:**
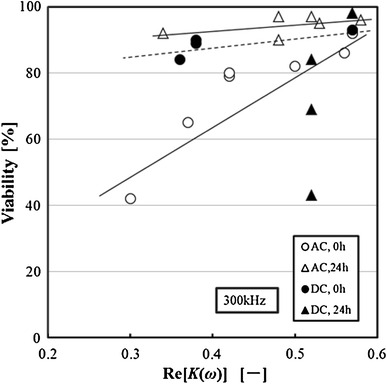
Relation between viability and Re[*K(ω)*] of 3-2H3 cells

To evaluate the cell activity by measuring the Re[*K(ω)*], the relation between the viability and Re[*K(ω)*] of the exposed cells in the DC electric field or the AC electric field was examined. The experimental results are shown in Fig. 13. The Re[*K(ω)*] of the cells after the AC electric field stress in this figure represents the value at 300 kHz. The viability of the cells exposed to the AC electric field decreased by decreasing Re[*K(ω)*]. Therefore, the correlation between the viability and the Re[*K(ω)*] of the exposed cells in the AC electric field was clarified. However, when the cells were cultivated for 24 h the viability was restored to more than 90 %, but the recovery of the Re[*K(ω)*] were low.

On the other hand, the viability of the cells exposed to the DC electric field decreased slightly by decreasing the Re[*K(ω)*]. The viability of the cells decreased significantly though the Re[*K(ω)*] were restored to 0.5 or more when the cells were cultivated for 24 h.

### Relation between growth activity and dielectric property

**Fig. 14:**
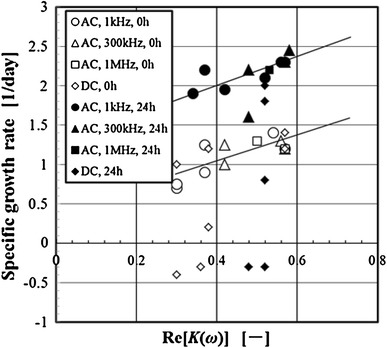
Relation between Re[*K(ω)*] and specific growth rate of 3-2H3 cells

To evaluate the cell activity by measuring the Re[*K(ω)*], the relation between the specific growth rate and the Re[*K(ω)*] of the exposed cells in the DC electric field or the AC electric field was examined. The experimental results are shown in Fig. 14. The experimental data of each voltage mode and frequency are the results provided under the condition of various applied voltage in Fig. 10. The Re[*K(ω)*] of the cells after the AC electric field stress in this figure represents the value at 300 kHz.

Therefore, the correlation between the specific growth rate and Re[*K(ω)*] of the exposed cell to the AC electric field was clarified. On the other hand, there was no correlation between the specific growth rate and Re[*K(ω)*] of the exposed cell to the DC electric field. The specific growth rate of the cells exposed to the DC electric field decreased by increasing the electric field strength. The tendency was significantly stronger than the tendency in the cells exposed to the AC electric field. In the DC electric field, the specific growth rate of the cells decreased remarkably because of influences such as the electrode reaction, the elution of metal ion, the generation of hydrogen peroxides, the pH changes, etc. We were not able to elucidate the cause of adverse effects only in the experimental results. Since the specific growth rate of the cells exposed to the DC electric field was not the only immediate adverse effect of the electric field, it was different from the specific growth rate of the cells exposed to the AC electric field. In the cells exposed to the DC electric field, it was considered that there was not a correlation between Re[*K(ω)*] and the viability or the growth activity.

## Conclusion

To evaluate the cell activity by measuring the Re[*K(ω)*], the relation between the cell activity and the dielectric property Re[*K(ω)*] of the cells exposed to the electric field was examined. The viability of the cells exposed to the DC electric field decreased slightly, but both the Re[*K(ω)*] and the specific growth rate drastically decreased. As for the cells after the cultivation, the Re[K(ω)] were restored but the viability and the specific growth rate decreased drastically, and the relation between the Re[*K(ω)*] and the specific growth rate was not proved. It was difficult to evaluate the growth activity of the cells exposed to the DC electric field by the Re[*K(ω)*]. On the other hand, the relations between the cell viability, the growth activity, and the Re[*K(ω)*] in the cells exposed to the AC electric field were obtained. The growth activity of the cells exposed to the AC electric field could be evaluated by the Re[*K(ω)*]. Furthermore, it was found that the adverse effects of the AC electric field on the cell viability and the growth activity were smaller than DC electric field.

## List of symbols

*E*: Effective value of electric field intensity (V/m)
*K*(*ω*): Clausius–Mossotti factor (dimensionless)
*R*: Particles radius (m)
*V*_*0*_: Applied voltage (V)
*X*: Cell concentration (cells/mL)
*z*: Distance between cell and plate electrode (m)
*z*_*min*_: Distance of electrode spacing (m)
*ε*: Dielectric permittivity (F/m)
*ρ*: Density (kg/m^3^)
*σ*: Electrical conductivity (S/m)
*ω*: Angular frequency (rad/s)

## Subscripts

0: Initial
*c*: Cell
*m*: Medium
*N*: Nth days
*V*: Viable cell
*NV*: Nonviable cell

## References

[1] Crane JS, Pohl HA (1968). A study of living and dead yeast cells using dielectrophoresis. J Electrochem Soc.

[2] Manson BD, Townsley PM (1970). Dielectrophoretic separation of cells. Can J Microbiol.

[3] Pohl HA, Kaler K (1979). Continuous dielectrophoretic separation of cells mixtures. Cell Biophys.

[4] Markx GH, Talary MS, Pething R (1994). Separation of viable and non-viable yeast using dielectrophoresis. J Biotechnol.

[5] Markx GH, Pething R (1995). Dielectrophoretic separation of cells: continuous separation. Biotechnol Bioeng.

[6] Docosilis A, Kalogerakis N, Behie LA, Kaler KVIS (1997). A novel dielectrophoresis-based device for selective retention of viable cells in cell culture media. Biotechnol Bioeng.

[7] Abidin ZZ, Downes L, Markx GH (2007). Large scale dielectrophoretic construction of biofilms using textile technology. Biotechnol Bioeng.

[8] Abidin ZZ, Downes L, Markx GH (2007). Novel electrode structures for large scale dielectrophoretic separations based on textile technology. J Biotechnol.

[9] Suzuki M, Yasukawa T, Shiku H, Matsue T (2005). Separation of live and dead microorganisms in a micro-fluidic device by dielectrophoresis. Bunseki Kagaku.

[10] Hirota Y, Hakoda M, Wakizaka Y (2010). Separation characteristics of animal cells using a dielectrophoretic filter. Bioprocess Biosyst Eng.

[11] Becker FF, Wang XB, Huang Y, Pethig R, Vykoukal J, Gascoyne PRC (1995). Separation of human breast cancer cells from blood by differential dielectric affinity. Proc Natl Acad Sci USA.

[12] Yang J, Huang Y, Wang XB, Becker F, Gascoyne PRC (2000). Differential analysis of human leukocytes by dielectrophoretic field flow fractionation. Biophy J.

[13] Wakizaka Y, Hakoda M, Shiragami N (2004). Effect of electrode geometry on dielectrophoretic separation of cells. Biochem Eng J.

[14] Fuhr G, Glasser H, Muller T, Schnelle T (1994). Cell manipulation and cultivation under a.c electric field influence in highly conductive culture medium. Biochim Biophys Acta.

[15] Docoslis A, Kalogerakis N, Behie LA (1999). Dielectrophoretic forces can be safely used to retain viable cells in perfusion cultures of animal cells. Cytotechnology.

[16] Foster KR, Sauer FA, Schwan HP (1992). Electrorotation and levitation of cells and colloidal particles. Biophys J.

[17] Kaler KVIS, Xie JP, Jones TB, Paul R (1992). Dual-frequency dielectrophoretic levitation of canola protoplasts. Biophys J.

[18] Hakoda M, Hachisu T, Wakizaka Y, Mii S, Kitajima N (2005). Development of a method to analyze single cell activity by using dielectrophoretic levitation. Biotechnol Prog.

[19] Hirota Y, Hakoda M (2010). Relationship between dielectric characteristic by DEP levitation and differentiation activity for rat mesenchymal stem cells. J Inst Electrostat Jpn.

[20] Hirota Y, Hakoda M (2011). Relationship between dielectric characteristic by DEP levitation and differentiation activity for stem cells. Key Eng Mater.

[21] Kaler KVIS, Jones TB (1990). Dielectrophoretic spectra of single cells determined by feedback-controlled levitation. Biophys J.

